# GC/MS^*n*^ analysis of the crude reaction mixtures from Friedel–Crafts acylation: Unambiguous identification and differentiation of 3‐aroylbenzofurans from their 4‐ and 6‐regioisomers

**DOI:** 10.1002/rcm.9082

**Published:** 2021-05-05

**Authors:** Michela Begala, Michele Mancinelli, Elias Quezada, Giovanna Lucia Delogu

**Affiliations:** ^1^ Department of Life and Environmental Sciences University of Cagliari, Cittadella Universitaria s.p.8, 09042 Monserrato Cagliari Italy; ^2^ Department of Industrial Chemistry “Toso Montanari” University of Bologna Viale del Risorgimento 4 Bologna 40136 Italy; ^3^ Department of Organic Chemistry University of Santiago de Compostela Santiago de Compostela 15782 Spain

## Abstract

**Rationale:**

3‐Aroylbenzofurans and their 2‐nitrophenyl derivatives constitute fundamental intermediates for the synthesis of target compounds with pharmaceutical properties. However, their preparation via the Friedel–Crafts acylation of 2‐phenylbenzofurans, using Lewis acid as catalyst, often leads to mixtures of regioisomeric aroylbenzofurans that can be challenging to distinguish, thus preventing the reaction characterization.

**Method:**

We report a method for the unambiguous identification and differentiation of the desired 3‐benzoyl isomers from their 4‐ and 6‐regioisomers in a crude reaction mixture using gas chromatography coupled to multiple‐stage mass spectrometric (GC/MS^*n*^) analysis performed in collision‐induced dissociation (CID) mode.

**Results:**

Upon electron ionization, each set of isomers displayed nearly identical mass spectra. MS^*n*^ revealed fragmentation patterns that varied in the location of the benzoyl group on the benzofuran scaffold: CID experiments performed on the molecular ion allowed the distinction of the 3‐acyl isomers from the 4‐ and 6‐regioisomers; CID experiments on the [M − Ar]^+^ ion allowed the distinction of the 4‐benzoyl from the 6‐benzoyl regioisomer, when the nitro group is located on the 2‐phenyl ring. Moreover, the unusual loss of OH^•^ radical allowed ascertaining the position of the nitro group in 3‐acyl regioisomers bearing the NO_2_ group. The origin of the diagnostic OH^•^ loss was investigated through MS^*n*^ experiments using ^18^O‐labelled 3‐benzoyl derivatives.

**Conclusions:**

The method allows the rapid characterization of crude reaction mixtures of benzoylbenzofurans using solely GC/MS^*n*^ analysis, simplifying the workflow of extensive isolation and purification for structure elucidation.

## 1 INTRODUCTION

**SCHEME 1:**
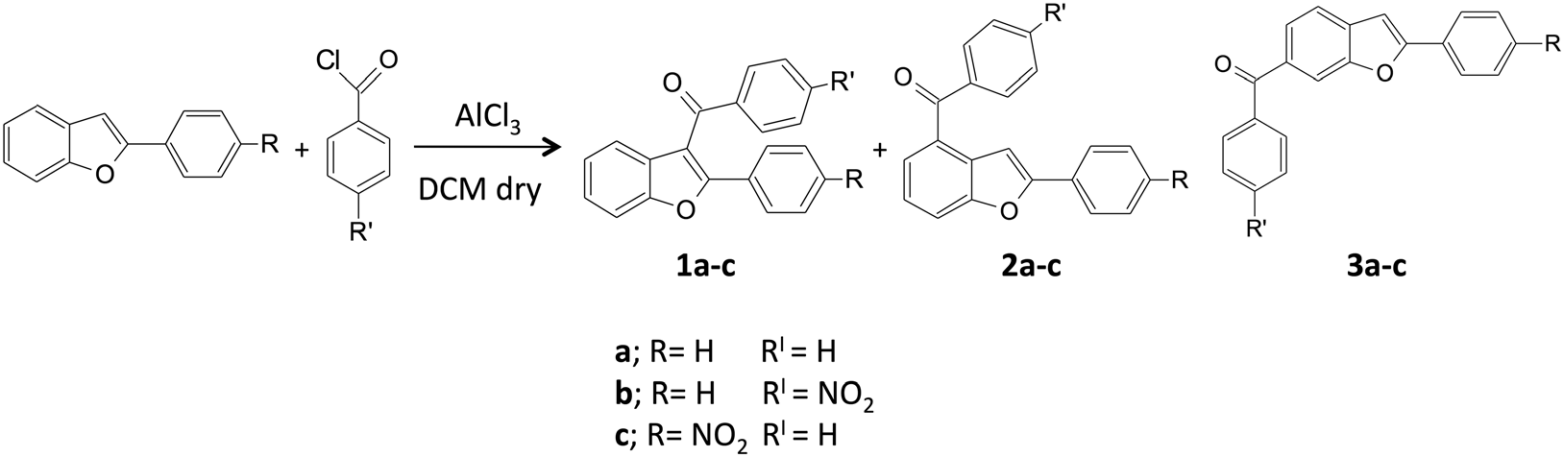
Mixtures of regioisomers obtained by Friedel–Crafts acylation of 2‐phenylbenzofurans

**FIGURE 1:**
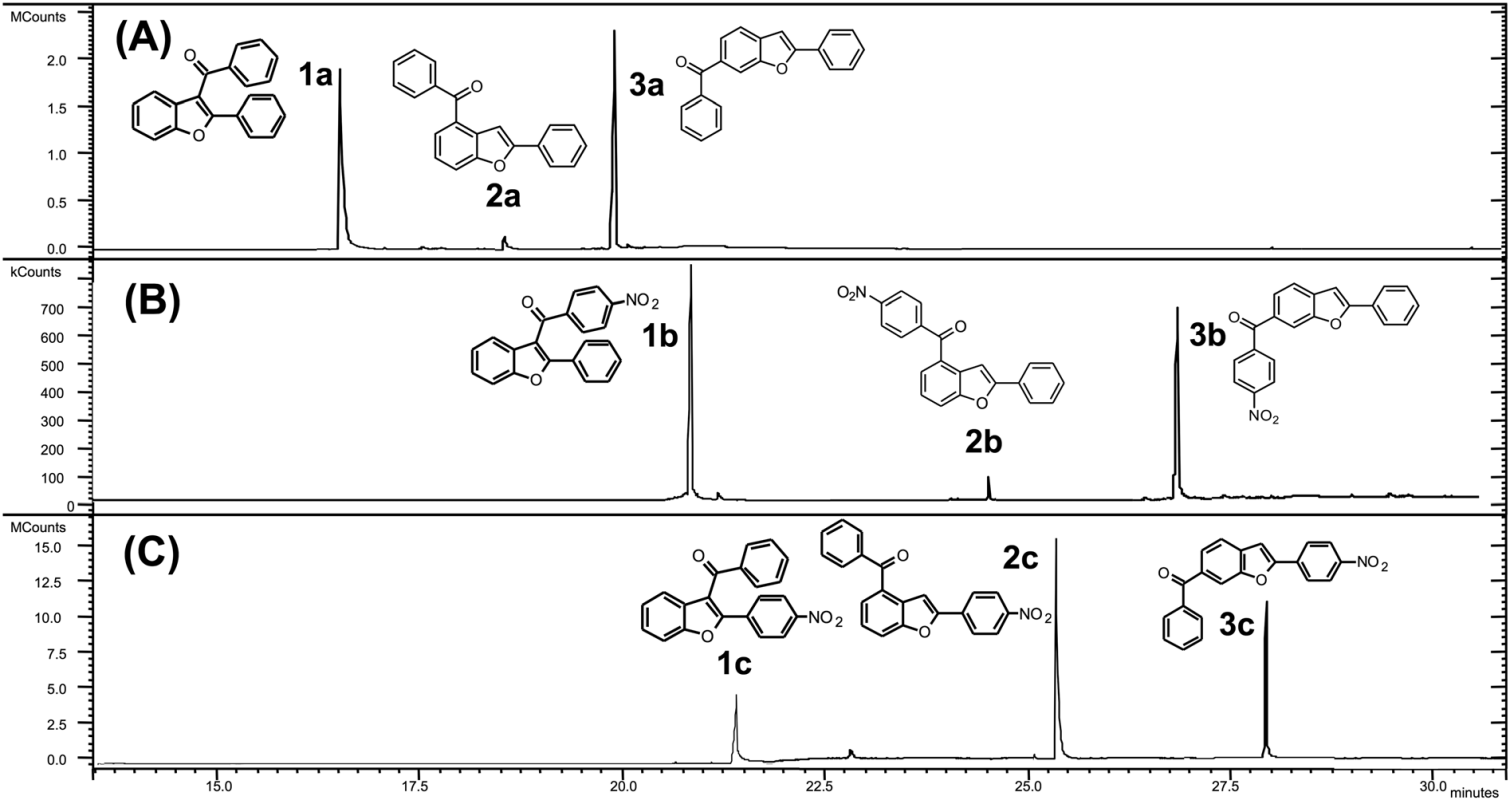
GC separation of the 3‐benzoylbenzofurans **1a**–**c** from their 4‐ and 6‐benzoyl regioisomers **2a**–**c** and **3a**–**c**

3‐Aroylbenzofurans represent an important class of heterocyclic compounds in current pharmaceuticals use or development.^1^, ^2^, ^3^, ^4^ As a result, various methods for the synthesis of 3‐aroylbenzofurans have been reported in the literature. The Friedel–Crafts acylation of aromatic compounds is one of the most widely used chemical reactions for the preparation of aromatic ketones at both academic and industrial levels.^5^ However, during the Friedel–Crafts aroylation of 2‐substituted benzofurans, various positions of the benzofuran ring are also acylated.^6^ More specifically, although aroylation occurs predominantly at the 3‐position, for most aroyl chlorides aroylation also occurs at the 6‐position and to a lesser extent at the 4‐position (Scheme 1; Figures 1A and 1B).^7^ A different regioisomer distribution is observed in the acylation of benzofurans deactivated by a nitro group on the 2‐phenyl ring (Figure 1C). In this case, Friedel–Crafts acylation leads to a mixture of regioisomers where the expected 3‐acyl isomer is formed as a minor product, reasonably because electron‐withdrawing groups attenuate the reactivity and influence position selectivity.^8^

Monitoring the reaction progress is of great interest in modern organic synthesis and drug research. However, thin‐layer chromatography is often ineffective for the analysis of complex reaction mixture, such as that of various benzoylbenzofuran regioisomers. Moreover, the purification and isolation of each benzoyl isomer from their mixtures is often a very laborious process that requires many manual steps to generate sufficiently pure compounds for identification and reaction characterization. In a worst case, the minor component cannot be separated.^9^ Hence, the need for sensitive methods that can rapidly identify benzoylbenzofuran regioisomers becomes crucial from this standpoint, especially for 3‐benzoyl‐2‐phenylbenzofurans containing nitro groups, as they could provide convenient intermediates in the preparation of more complicated compounds.^10^, ^11^, ^12^

In this paper, we report the gas chromatography coupled to multiple‐stage mass spectrometric (GC/MS^*n*^) analyses of three series of isomeric mixtures of benzoylbenzofurans obtained via Friedel–Crafts acylation of 2‐phenylbenzofurans with benzoyl chlorides (Figure 1 and Scheme 1), aiming to distinguish and to identify, in the crude reaction mixtures, the 3‐benzoyl isomers **1a**–**c** from their 4‐ and 6‐regioisomers **2a**–**c** and **3a**–**c**, respectively. For this aim, MS^*n*^ experiments, performed in collision‐induced dissociation (CID) mode, were carried out using a quadrupole ion trap analyzer.^13^

## 2 EXPERIMENTAL

### 2.1 MS analysis

All experiments were performed with a Varian Saturn 2000 ion trap mass spectrometer, operating under electron ionization (EI) conditions (electron energy, 70 eV; emission current, 20 mA; ion trap temperature, 220°C; manifold temperature, 80°C; automatic gain control target, 21 000) with the ion trap operating in scan mode (scan range *m*/*z* 40–500 at a scan rate of 1 scan/s), coupled with a Varian 3800 gas chromatograph (Varian, Walnut Creek, CA).

CID experiments were carried out using helium as the collision gas (gas purity was 99.9999%). For MS^*n*^ experiments the supplementary rf voltage (45–55 V) was varied in such a way that the relative abundance of the surviving precursor ions was 5%–15%. An isolation time of 10 ms, an excitation time of 40 ms and an isolation width of 1 *m*/*z* for the precursor ion were used. Each MS/MS spectrum was an average of five scans.

Compounds under investigation (1 μL aliquots of 1.0 × 10^−5^ M solutions in dichloromethane) were introduced into the GC inlet. To perform the GC separation, an Agilent J&W VF‐5ms low‐bleed/MS GC capillary column (30 m, 0.25 mm i.d., 0.25 μm film thickness; Agilent Technologies Inc., Wilmington, DE, USA) was used with a flow of 1.0 mL/min. The oven temperature was programmed from 80°C (held for 5 min) to 280°C at 20°C/min (held for 5 min); this was increased to 300°C at 20°C/min (held for 5 min). The temperature was then ramped to 350°C at 20°C/min. The transfer line was maintained at 180°C and the injector port (30:1 split) at 290°C. MS^*n*^ analyses were replicated five times. The relative standard deviations of the relative peak intensities were below 15% (Tables S1–S18); in the measurements, very small peak are usually excluded and we used an intensity cutoff of 2%.

### 2.2 Materials and reagents

All reagents and solvents were purchased from Sigma‐Aldrich S.r.l. (Milan, Italy) and Carlo Erba Reagents S.r.l. (Milan, Italy) and used without further purification.

The crude reaction mixtures of regioisomers **1a**–**3a**, **1b**–**3b** and **1c**–**3c** were prepared via direct Friedel–Crafts acylation of the respective 2‐phenylbenzofuran with benzoyl chloride using AlCl_3_ as Lewis acid in anhydrous dichloromethane (Scheme 1).^8^

3‐Benzoyl‐2‐phenyl‐*d*_5_‐benzofurans **1a*d***_**5**_–**3a*d***_**5**_ were synthesized in the same way starting from 2‐phenylbenzofuran‐*d*_5_ and benzoyl chloride.

Reference compounds **1a**, **1b** and **1c** were prepared as reported in our previous works.^14^, ^15^

2‐Phenylbenzofuran, 2‐phenylbenzofuran‐*d*_5_ and 2‐(4‐nitrophenyl)benzofuran were prepared starting from 2‐hydroxybenzyltriphenylphosphomium bromide and benzoyl chloride, benzoyl chloride‐*d*_5_ or 4‐nitrobenzoyl chloride, respectively, in toluene and Et_3_N using the procedure described in previous works.^14^, ^16^, ^17^

3‐[^18^O]‐Benzoyl‐2‐(4‐nitrophenyl)benzofuran (**1c[** ^**18**^ **O]**) was prepared analogously to **1c** starting from 2‐(4‐nitrophenyl)benzofuran and [^18^O]‐benzoyl chloride.

[^18^O]‐Benzoyl chloride was prepared by acid‐catalyzed hydrolysis (HCl/H_2_^18^O) of benzonitrile and treatment of [^18^O]‐benzoic acid with thionyl chloride as described by Wnuk and co‐workers.^18^

Compounds **1d** and **1e** were obtained by the reaction of 2‐(4‐methoxybenzyloxy)benzyltriphenylphosphomium bromide and 4‐nitrobenzoyl chlorides in the presence of Et_3_N in toluene.^15^

All benzoylbenzofuran isomers and the labeled derivatives were isolated and characterized using ^13^C NMR and ^1^H NMR as described in the supporting information and in previous reports.^14^, ^15^, ^16^ Isomers **2a**, **2a*d***_**5**_, **2b** and **2c** were further purified using high‐performance liquid chromatography.

## 3 RESULTS AND DISCUSSION

### 3.1 Separation and differentiation of isomers **1a**–**3a**

**FIGURE 2:**
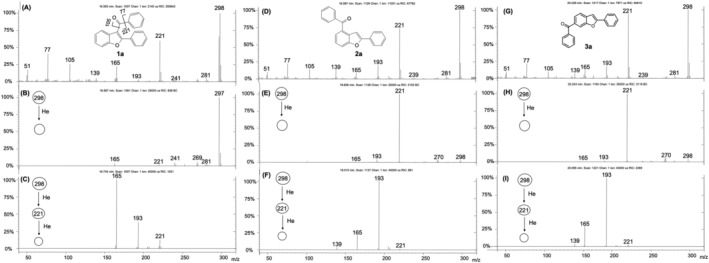
EI mass spectrum (A), MS^2^ of *m*/*z* 298 (B) and MS^3^ of *m*/*z* 221 (C) of the 3‐acyl isomer **1a**; EI mass spectrum (D), MS^2^ of *m*/*z* 298 (E) and MS^3^ of *m*/*z* 221 (F) of the 4‐acyl isomer **2a**; EI mass spectrum (G), MS^2^ of *m*/*z* 298 (H) and MS^3^ of *m*/*z* 221 (I) of the 6‐acyl isomer **3a**

**FIGURE 3:**
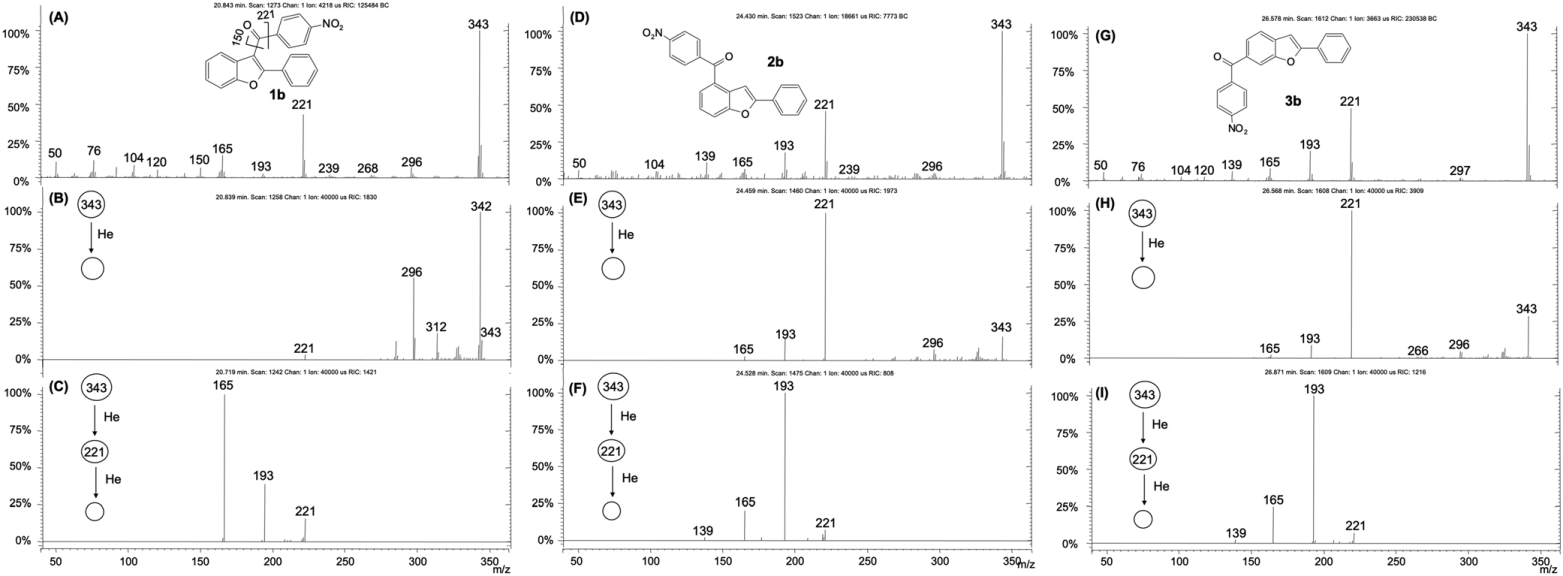
EI mass spectrum (A), MS^2^ of *m*/*z* 343 (B) and MS^2^ of *m*/*z* 221 (C) of the 3‐acyl isomer **1b**; EI mass spectrum (D), MS^2^ of *m*/*z* 343 (E) and MS^3^ of *m*/*z* 221 (F) of the 4‐acyl isomer **2b**; EI mass spectrum (G), MS^2^ of *m*/*z* 343 (H) and MS^3^ of *m*/*z* 221 (I) of the 6‐acyl isomer **3b**

The total ion current chromatogram of the reaction products from the Friedel–Crafts acylation of 2‐phenylbenzofuran with benzoyl chloride is reported in Figure 1A. The three isomers so obtained are well separated, and the ion intensities of the isomers are different from each other. After purification, we found that acylation leads predominantly to the 3‐acyl isomer **1a** together with traces of the 4‐benzoyl isomer **2a** and a larger amount of the 6‐benzoyl isomer **3a**. All isomers exhibit very similar EI mass spectra (Figures 2A, 2D and 2G). The main fragmentation pathways lead to ions at *m*/*z* 221, 105 and 77. Only the mass spectrum of isomer **1a** shows a unique diagnostic ion at *m*/*z* 297 owing to the loss of radical hydrogen (Figure 2A). The hydrogen loss is a typical behavior of 3‐acyl isomers as it is the result of the interaction between the 3‐carbonyl group with the neighboring phenyl ring in the 2‐position.^16^ In the case of isomers **2a** and **3a**, the [M − H]^+^ ion is not observed reasonably because the distances between the carbonyl group and the 2‐phenyl ring are much greater than in the 3‐benzoyl isomer **1a**, thus avoiding their interaction. MS^2^ experiments performed on the molecular ion at *m*/*z* 298 and on the *m*/*z* 221 ion of **1a**–**3a** evidenced further distinctive fragmentation patterns making the 3‐acyl isomer (**1a**) well recognizable from the other two (Figure 2). On the contrary, the 4‐benzoyl isomer (**2a**) and the 6‐benzoyl isomer (**3a**) remained indistinguishable. In the MS/MS spectrum of the *m*/*z* 298 ion, the diagnostic [M − H]^+^ ion (*m*/*z* 297) is even more favored for isomer **1a** (Figure 2B) and it is absent in the MS/MS spectra of **2a** and **3a** (Figures 2E and 2H). Instead, **2a** and **3a** display the ion at *m*/*z* 221 as the base peak, which is particularly weak for the 3‐benzoyl isomer **1a**. This ion corresponds for all isomers to the loss of radical C_6_H_5_^•^ from the benzoyl group, as supported by the mass shift to *m*/*z* 226 in the spectra of the deuterium analogues **1a*d***_**5**_–**3a*d***_**5**_ (Figures S1 and S2). Therefore, in the structure of the fragment ion at *m*/*z* 221, the positional information of the carbonyl group on the benzofuran scaffold is still preserved. Accordingly, further differences in ion abundances were evidenced in the MS^3^ spectra of the *m*/*z* 221 ions (Figures 2C, 2F and 2I): although all isomers exhibit the loss of CO molecules (*m*/*z* 193) followed by the typical abstraction of further CO from the 2‐phenylbenzofuran nucleus (*m*/*z* 165),^19^ only for isomer **1a** does the ion at *m*/*z* 165 constitute the base peak (Figure 2C), whereas the ion at *m*/*z* 193 is the major peak for isomers **2a** (Figure 2F) and **3a** (Figure 2I).

### 3.2 Separation and differentiation of isomers **1b**–**3b**

The total ion current chromatogram of the Friedel–Crafts acylation of 2‐phenylbenzofuran with 4‐nitrobenzoyl chloride is shown in Figure 1B. The most abundant peak corresponds to the 3‐acyl isomer **1b**, whereas the lower intensity peaks at *t*_R_ of 24.45 and 26.54 min correspond to the 4‐ and 6‐acyl isomers **2b** and **3b**, respectively. The mass spectra of isomeric compounds **1b**–**3b**, despite some minor differences in relative abundances of fragment ions, are nearly identical (Figures 3A, 3D and 3G). Each isomer decomposes by the typical alpha cleavage to the carbonyl group, to afford the loss of the 4‐nitrophenyl group (*m*/*z* 221) and the 4‐nitrobenzoyl cation (*m*/*z* 150). Conversely, the MS/MS experiments performed on the molecular ions at *m*/*z* 343 are effective for differentiating the 3‐benzoyl isomer **1b** from the other two, **2b** and **3b** (Figures 3B, 3E and 3H). Under this condition the 3‐benzoyl isomer **1b** leads to the ions at *m*/*z* 342 and 296 attributed to the [M − H]^+^ and the [M − H − NO_2_]^+•^ ions, respectively. Both ions are particularly intense in the mass spectrum of **1b**, whereas they are weak in the mass spectra of isomers **2b** and **3b**, reasonably because the carbonyl group is too far from the 2‐phenyl ring to promote the initial H• loss (discussed above). Unlike **1b**, both isomers **2b** and **3b** exhibit the ion at *m*/*z* 221 as base peak. MS^3^ experiments on the *m*/*z* 221 ion allow for additional differentiation of **1b** from **2b** and **3b** (Figures 3C, 3F and 3I).

### 3.3 Separation and differentiation of isomers **1c**–**3c**

**FIGURE 4:**
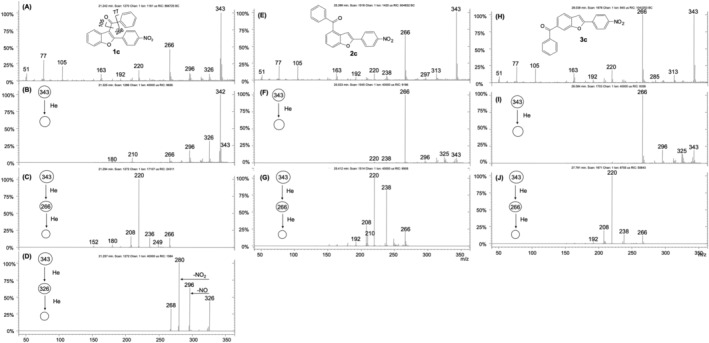
EI mass spectrum (A), MS^2^ of *m*/*z* 343 (B), MS^3^ of *m*/*z* 266 (C) and MS^2^ of *m*/*z* 326 (D) of the 3‐acyl isomer **1c**; EI mass spectrum (E), MS^2^ of *m*/*z* 343 (F) and MS^3^ of *m*/*z* 266 (G) of the 4‐acyl isomer **2c**; EI mass spectrum (H), MS^2^ of *m*/*z* 343 (I) and MS^3^ of *m*/*z* 266 (J) of the 6‐acyl isomer **3c**

**SCHEME 2:**
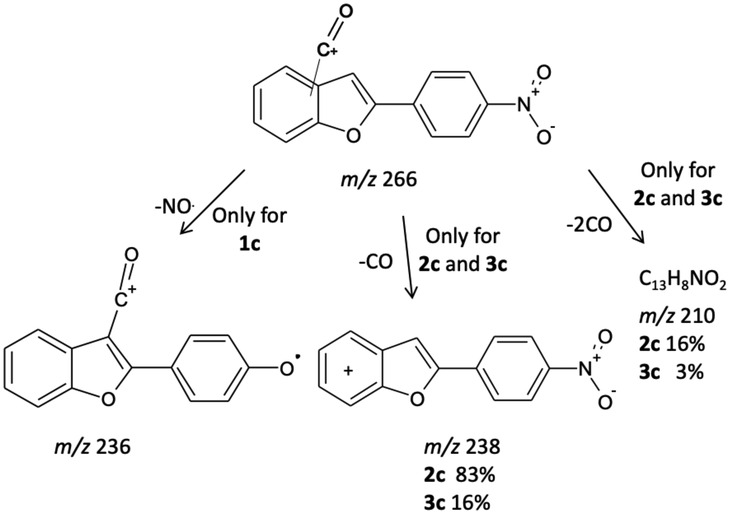
MS^n^ fragmentation pathways for compound **1c** **^18^O**

The Friedel–Crafts acylation of 2‐(4‐nitrophenyl)benzofuran with benzoyl chloride leads to a mixture of regioisomers where the desired 3‐acyl derivative is formed as a minor product (Figure 1C). The EI mass spectra of the nitrobenzoyl isomers **1c**–**3c** (Figures 4A, 4E and 4H) show a rather similar mass fragmentation behavior, i.e. the formation of the benzoyl cation at *m*/*z* 105, and the characteristic loss of the phenyl ring (*m*/*z* 266). Under MS/MS experiments the molecular ions (*m*/*z* 343) of regioisomers **2c** and **3c** behave quite differently from that of the 3‐acyl isomer **1c** as they exhibit the loss of radical C_6_H_5_^•^ from the benzoyl group as the main peak (*m*/*z* 266). Interestingly, only in this series of isomers can the 4‐acyl isomer be differentiated from the 6‐acyl isomer through MS^3^ experiments on the *m*/*z* 266 ion: both isomers exhibit the loss of nitrogen dioxide (NO_2_) to form the *m*/*z* 220 ion as the base peak, whereas only for the 4‐acyl isomer **2c** are the ions at *m*/*z* 238 (83%) and 210 (16%) particularly intense allowing differentiation from the 6‐acyl isomer **3c** (*m*/*z* 238, 16%; *m*/*z* 210, 3%) (Figures 4G and 4J; Scheme 2). The statistical significance of differences between the relative intensities of ions at *m*/*z* 210 and 238 between **2c** and **3c** was confirmed by Welch's *t*‐test (*p* < 0.0001).

The 3‐acyl isomer **1c** can be easily identified and differentiated from **2c** and **3c** on the bases of MS/MS experiments on the molecular ion (Figures 4B, 4F and 4I). Under these conditions, the formation of the *m*/*z* 266 ion, favored for **2c** and **3c**, is practically suppressed. Instead, the *m*/*z* 343 ion exhibits the ion at *m*/*z* 342 as the main peak and the ion at *m*/*z* 326 due to the elimination of OH^•^ radical (see below).

The lack of the [M − OH]^+^ ion in the MS/MS spectrum of the nitro isomer **1b** (Figure 3B) clearly suggests that, when the NO_2_ group is on the 2‐phenyl ring, it may play an essential role in the hydroxyl radical loss. A previous study showed that the loss of OH^•^ occurred in the mass spectrum of 3‐benzoyl‐2‐phenylbenzofuran bearing nitro groups on both phenyl rings.^16^ However, it was not possible to verify if the source of the OH^•^ loss was the nitro group, through a chalcone‐like mechanism,^20^ the oxygen of the carbonyl or both groups. Since the pathways that lead to the loss of OH remained an opened question in the previous study, further experiments were undertaken in the present work.

**SCHEME 3:**
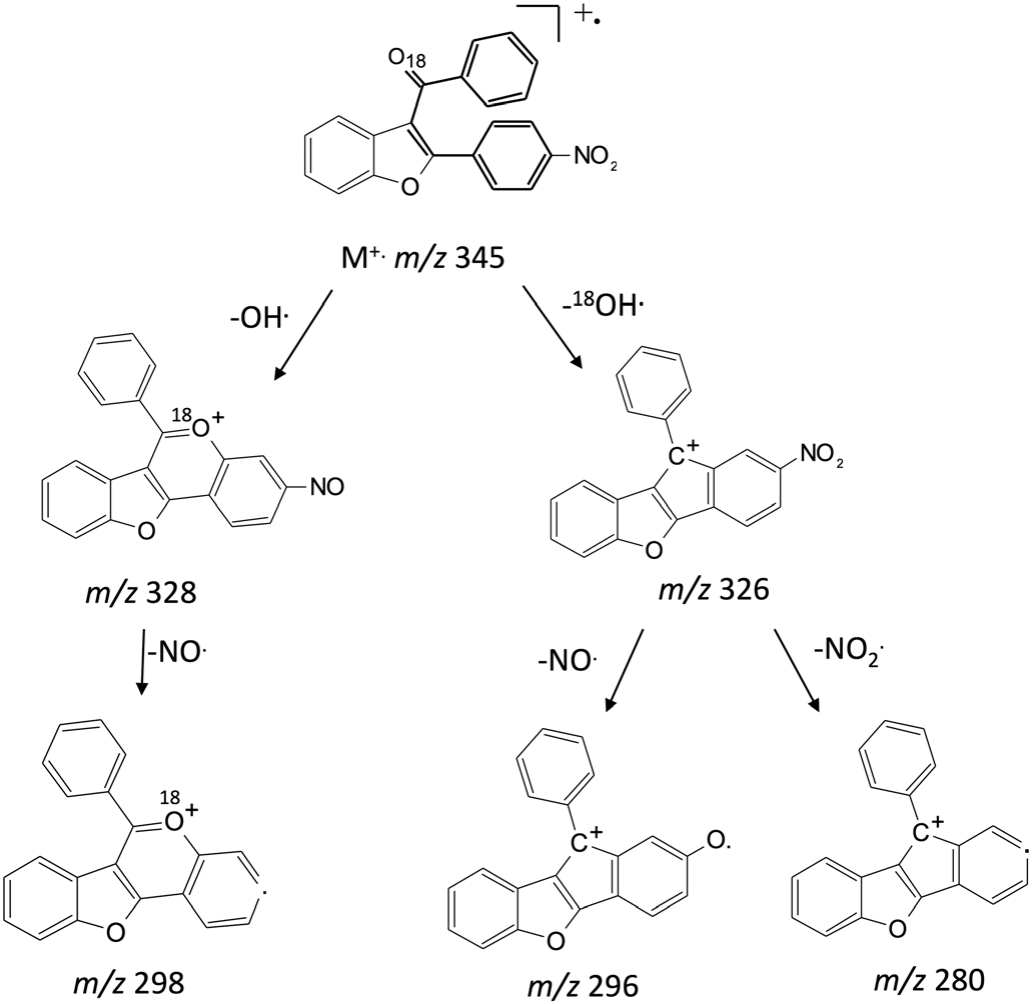
MS^*n*^ fragmentation pathways for compound **1c** ^**18**^ **O**

MS^3^ experiments performed on the [M − OH]^+^ ion from isomer **1c** (*m*/*z* 326) activate, together with the loss of NO^•^ (*m*/*z* 296), the loss of NO_2_^•^ (*m*/*z* 280), which demonstrates that the oxygen involved in the OH^•^ loss arises, at least in part, from the carbonyl group (Figure 4D; Scheme 3). This behavior is of particular interest as there are only few reports of the involvement of a carbonyl oxygen atom in the formation of dehydroxylated ions.^21^, ^22^, ^23^, ^24^

**FIGURE 5:**
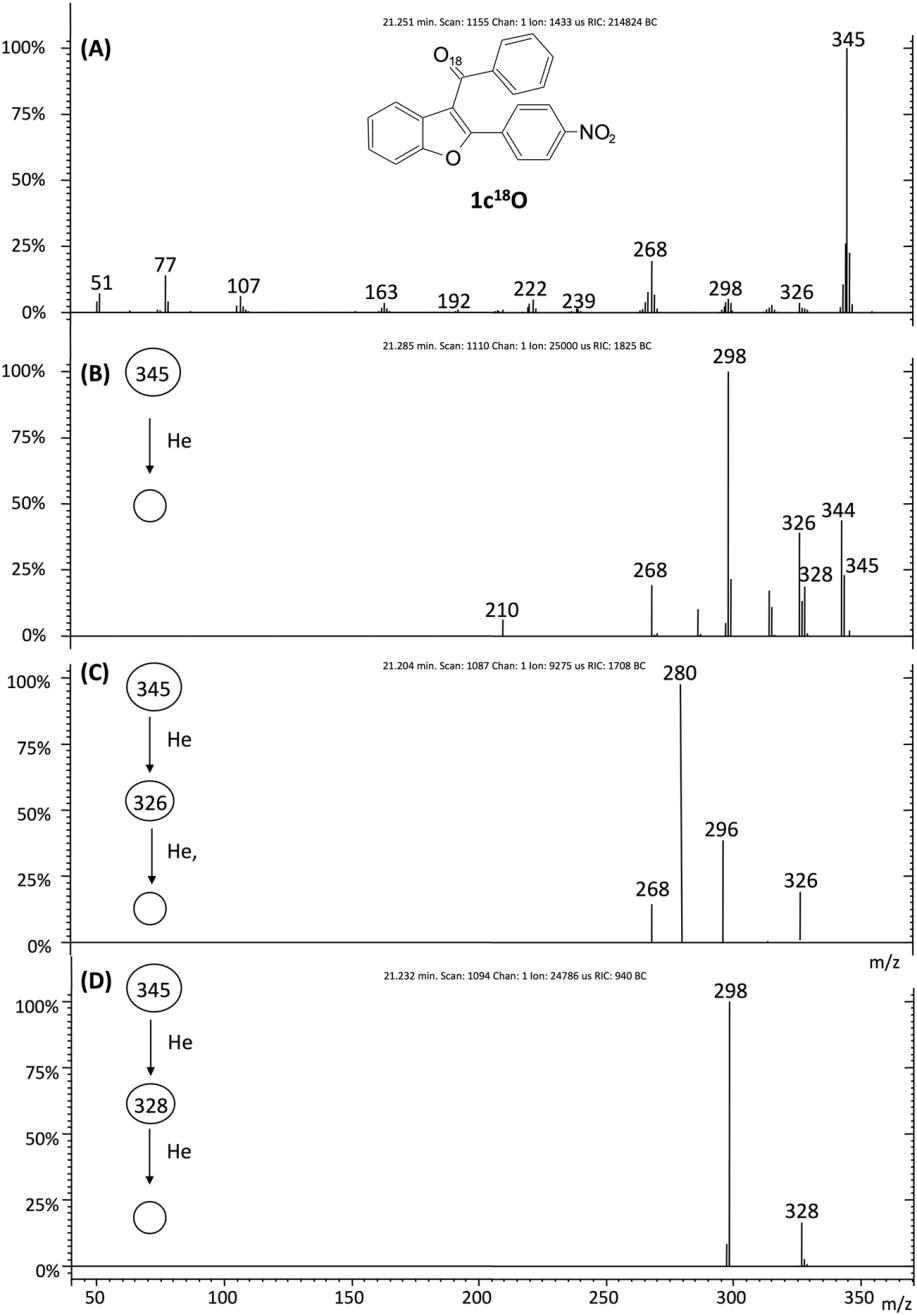
EI mass spectrum (A), MS^2^ of *m*/*z* 345 (B), MS^3^ of *m*/*z* 326 (C) and MS^3^ of *m*/*z* 328 (D) of the labelled 3‐acyl isomer **1c** ^**18**^ **O**

Some experiments were also performed on the analogous 3‐benzoyl‐2‐(4‐nitrophenyl)benzofuran **1c[** ^**18**^ **O]** labeled with ^18^O at the carbonyl group (Figure 5; Scheme 3). The MS^2^ experiments performed on the molecular ion at *m*/*z* 345 showed the formation of [M − ^18^OH]^+^ ion at *m*/*z* 326 and of [M − OH]^+^ ion at *m*/*z* 328 (Figure 5B). These data prove that the OH^•^ loss arises from the oxygen of both groups. This conclusion is corroborated by the losses of NO^•^ and NO_2_^•^ from the [M − ^18^OH]^+^ ion (*m*/*z* 326), and by the loss of NO^•^ from the [M − OH]^+^ ion (*m*/*z* 328) under MS^3^ experiments (Figures 5C and 5D; Scheme 3). Interestingly, the intensity of the *m*/*z* 326 ion is significantly higher than that of the *m*/*z* 328 ion (relative intensity 47% versus 15%), thus demonstrating that the oxygen of the carbonyl group contributes more significantly to the OH^•^ loss. However, when the source of OH^•^ loss is exclusively the carbonyl group, such as in the case of the unsubstituted compound **1a**, the intensity of the [M − OH]^+^ ion is very low (*m*/*z* 281, relative intensity 3%; Figure 2B). These data suggest that the OH^•^ loss is significantly increased by the presence of the nitro group on the 2‐phenyl ring, not only because the NO_2_ group can itself eliminate radical OH^•^, but also because it has an essential influence on the OH^•^ elimination from the carbonyl group.

**FIGURE 6:**
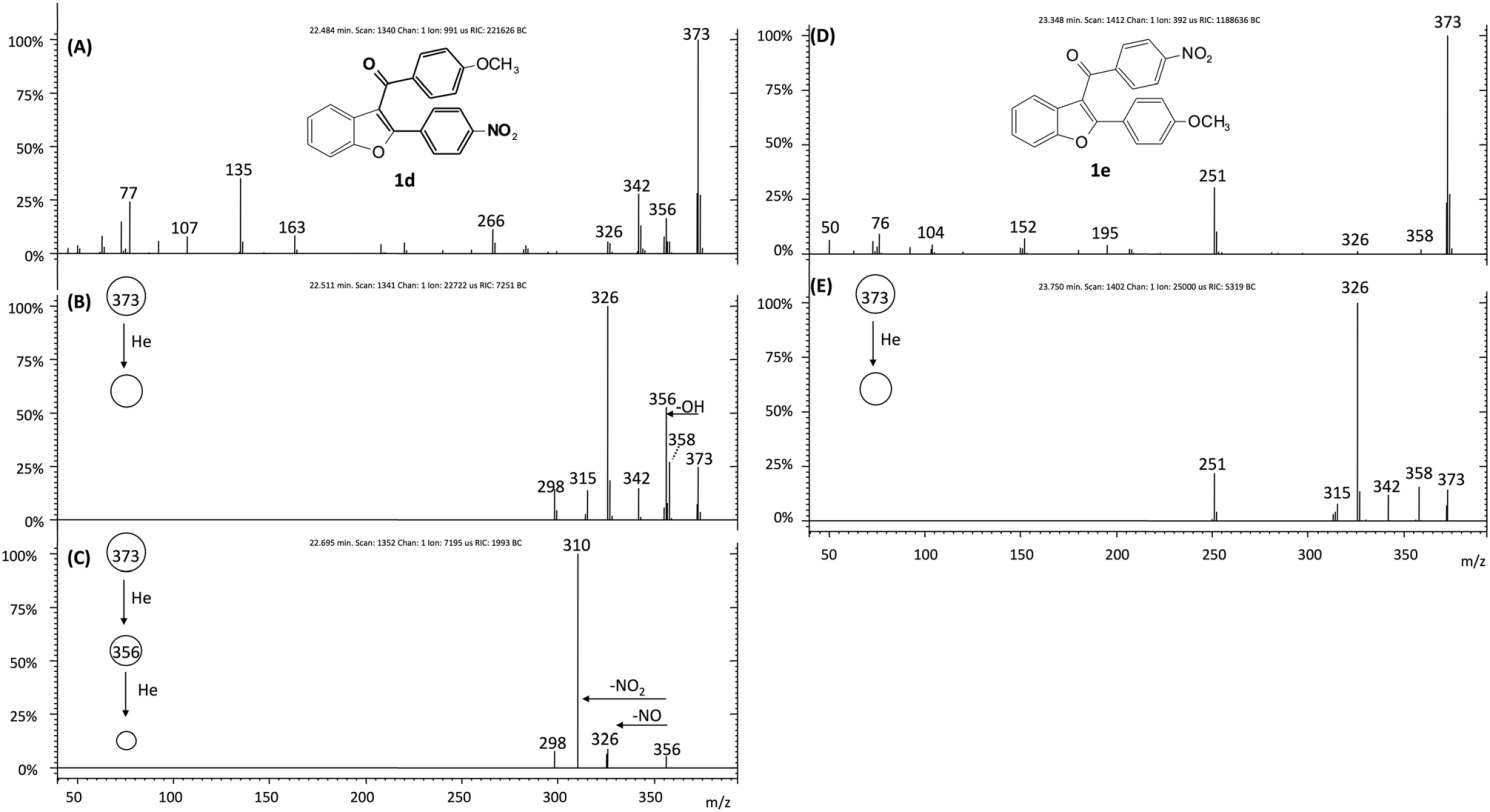
EI mass spectrum (A), MS^2^ of *m*/*z* 373 (B) and MS^3^ of *m*/*z* 356 (C) of the isomer **1d**; EI mass spectrum (D) and MS^2^ of *m*/*z* 373 (E) of the isomer **1e**

To show that the [M − OH]^+^ ion functions as a structurally diagnostic product ion for the identification of the 3‐benzoyl‐2‐(4‐nitrophenyl)benzofuran nucleus, we examined the mass spectra of two isomeric nitro derivatives **1d** and **1e** (Figure 6).^15^ Under MS/MS conditions only the 3‐(4‐methoxybenzoyl)‐2‐(4‐nitrophenyl)benzofuran **1d** (*m*/*z* 373) displays the loss of OH^•^ radical generating the ion at *m*/*z* 356 (Figure 6B) that further loses NO_2_^•^ under MS^3^ experiments to form ion at *m*/*z* 310 (Figure 6C). Conversely, the 2‐(4‐methoxyphenyl)‐3‐(4‐nitrobenzoyl)benzofuran **1e** does not show the OH^•^ loss (Figures 6D and 6E), unequivocally proving the lack of the 3‐benzoyl‐2‐(4‐nitrophenyl)benzofuran nucleus in its structure.

## 4 CONCLUSIONS

Regioisomeric mixtures of 3‐, 4‐ and 6‐benzoyl‐2‐phenylbenzofurans are obtained by Friedel–Crafts acylation of 2‐phenylbenzofurans. GC/MS can be used for the rapid separation and identification of 3‐benzoyl‐2‐phenylbenzofurans from their 4‐ and 6‐benzoyl regioisomers directly in the crude reaction mixtures without the need of reference standards. EI‐MS^*n*^ experiments on the molecular ions yield specific fragments which arise from the interaction of the 3‐benzoyl group with the neighbor 2‐phenyl ring, i.e. [M − H]^+^ and [M − OH]^+^ ions, nearly absent in the mass spectra of the other regioisomers. These diagnostic ions indicate that the benzoyl group is located in the 3‐position of the 2‐phenylbenzofuran skeleton. 3‐Acyl isomers containing nitro group in their skeleton display abundant [M − OH]^+^ ions (relative intensity > 40) only when the nitro group is located on the 2‐phenyl ring. We hypothesize that this behavior could be expanded to derivatives that possess the 4‐nitrochalcone (Ar&bond;[C&dbond;O]&bond;C&dbond;C&bond;C_6_H_4_NO_2_) structural fragment.

The 4‐ and 6‐benzoyl regioisomers, unsubstituted on the 2‐phenyl ring, show an identical mass spectrometric behavior, even under MS^*n*^ experiments. 4‐Benzoyl‐2‐nitrophenyl regioisomer **2c** displays a specific mass spectrometric behavior of the [M − Ar]^+^ fragment ion which allows the distinction from its 6‐benzoyl‐2‐nitrophenyl regioisomer **3c**.

5

### PEER REVIEW

The peer review history for this article is available at https://publons.com/publon/10.1002/rcm.9082.

## Supporting information


**Figure S1.** EI mass spectra of labelled regioisomers **1a*d***_***5***_ (A), **2a*d***_***5***_ (B), and **3a*d***_***5***_ (C).
**Figure S2.** MS/MS mass spectra of the ions at *m/z* 303 from labeled **1a*d***_***5***_ (A), **2a*d***_***5***_ (B), and **3a*d***_***5***_ (C).
**Figure S3.** EI mass spectra of [2 x ^18^O]‐benzoic acid (A), and of [^18^O]‐benzoyl chloride (B).
**Figure S4.** 600 MHz ^1^H NMR spectrum and 151 MHz ^13^C NMR spectrum of 4‐benzoyl‐2‐phenylbenzofuran (**2a**).
**Figure S5.** HSQC and COSY spectra of 4‐benzoyl‐2‐phenylbenzofuran (**2a**).
**Figure S6.** 600 MHz ^1^H NMR spectrum and 151 MHz ^13^C NMR spectrum of 6‐benzoyl‐2‐phenylbenzofuran (**3a**).
**Figure S7.** 600 MHz ^1^H NMR in CDCl_3_ spectrum and NOESY‐1D spectrum obtained by irradiating the signal of the hydrogen in position 3d of 6‐benzoyl‐2‐phenylbenzofuran (**3a**).
**Figure S8.** 600 MHz ^1^H NMR spectrum and 151 MHz ^13^C NMR spectrum of 3‐benzoyl‐2‐phenyl(d_5_)benzofuran (**1a*d***_***5***_).
**Figure S9.** 600 MHz ^1^H NMR spectrum and 151 MHz ^13^C NMR spectrum of 4‐benzoyl‐2‐phenyl(d_5_)benzofuran (**2a*d***_***5***_).
**Figure S10.** 600 MHz ^1^H NMR spectrum and 151 MHz ^13^C NMR spectrum of 6‐benzoyl‐2‐phenyl(d_5_)benzofuran (**3a*d***_***5***_).
**Figure S11.** 600 MHz ^1^H NMR spectrum and 151 MHz ^13^C NMR spectrum of 4‐(4‐nitrobenzoyl)‐2‐phenylbenzofuran (**2b**).
**Figure S12.** Top: 600 MHz COSY spectra of 4‐(4‐nitrobenzoyl)‐2‐phenylbenzofuran (**2b**). Bottom: 600 MHz ^1^H NMR in CDCl_3_ and NOESY‐1D spectra obtained by irradiating the signal of the hydrogen in position 3d of **2b**.
**Figure S13.** 600 MHz ^1^H NMR spectrum and 151 MHz ^13^C NMR spectrum of 6‐(4‐nitrobenzoyl)‐2‐phenylbenzofuran (**3b**).
**Figure S14.** 600 MHz ^1^H NMR in CDCl_3_ and NOESY‐1D spectra obtained by irradiating the signal of the hydrogen in position 3d of *6‐(4‐nitrobenzoyl)‐2‐phenylbenzofuran* (**3b**).
**Figure S15.** 600 MHz ^1^H NMR spectrum and 151 MHz ^13^C NMR spectrum of 4‐benzoyl‐2‐(4‐nitrophenyl)benzofuran (**2c**).
**Figure S16.** Top: 600 MHz COSY spectra of 4‐benzoyl‐2‐(4‐nitrophenyl)benzofuran (**2c**). Bottom: 600 MHz ^1^H NMR in CDCl_3_ and NOESY‐1D spectra obtained by irradiating the signal of the hydrogen in position 3d of **2c**.
**Figure S17.** 600 MHz ^1^H NMR spectrum and 151 MHz ^13^C NMR spectrum of 6‐benzoyl‐2‐(4‐nitrophenyl)benzofuran (**3c**).
**Figure S18.** 600 MHz ^1^H NMR in CDCl_3_ and NOESY‐1D spectra obtained by irradiating the signal of the hydrogen in position 3d of 6‐benzoyl‐2‐(4‐nitrophenyl)benzofuran (**3c**).
**Scheme S1.** Proposed mechanisms of elimination of radical OH^.^ from labeled **1c[** ^**18**^ **O]**.
**Table S1.** Repeatability of relative peak intensity of ions in MS^2^ spectrum of **1a**
**Table S2.** Repeatability of relative peak intensity of ions in MS^3^ spectra of **1a**
**Table S3.** Repeatability of relative peak intensity of ions in MS^2^ spectrum of **2a**
**Table S4.** Repeatability of relative peak intensity of ions in MS^3^ s spectrum of **2a**
**Table S5.** Repeatability of relative peak intensity of ions in MS^2^ spectrum of **3a**
**Table S6.** Repeatability of relative peak intensity of ions in MS^3^ spectrum of **3a**
**Table S7.** Repeatability of relative peak intensity of ions in MS^2^spectrum of **1b**
**Table S8.** Repeatability of relative peak intensity of ions in MS^3^ spectra of **1b**
**Table S9.** Repeatability of relative peak intensity of ions in MS^2^ spectrum of **2b**
**Table S10.** Repeatability of relative peak intensity of ions in MS^3^ spectrum of **2b**
**Table S11.** Repeatability of relative peak intensity of ions in MS^2^ spectrum of **3b**
**Table S12.** Repeatability of relative peak intensity of ions in MS^3^ spectrum of **3b**
**Table S13.** Repeatability of relative peak intensity of ions in MS^2^ spectrum of **1c**
**Table S14.** Repeatability of relative peak intensity of ions in MS^3^ spectrum of **1c**
**Table S15.** Repeatability of relative peak intensity of ions in MS^2^ spectrum of **2c**
**Table S16.** Repeatability of relative peak intensity of ions in MS^3^ spectrum of **2c**
**Table S17.** Repeatability of relative peak intensity of ions in MS^2^ spectrum of **3c**
**Table S18.** Repeatability of relative peak intensity of ions in MS^3^ spectrum of **3c**
