# Supplementary material for: GC/MSn analysis of the crude reaction mixtures from Friedel–Crafts acylation: Unambiguous identification and differentiation of 3‐aroylbenzofurans from their 4‐ and 6‐regioisomers
Source: Rapid Commun Mass Spectrom. 2021 May 5;35(11):e9082. doi: 10.1002/rcm.9082 (PMC8244086; doi:10.1002/rcm.9082)
Supplement: Supplementary file 1 — Figure S1. EI mass spectra of labelled regioisomers 1ad 5 (A), 2ad 5 (B), and 3ad 5 (C). Figure S2. MS/MS mass spectra of the ions at m/z 303 from labeled 1ad 5 (A), 2ad 5 (B), and 3ad 5 (C). Figure S3. EI mass spectra of [2 x 18O]‐benzoic acid (A), and of [18O]‐benzoyl chloride (B). Figure S4. 600 MHz 1H NMR spectrum and 151 MHz 13C NMR spectrum of 4‐benzoyl‐2‐phenylbenzofuran (2a). Figure S5. HSQC and COSY spectra of 4‐benzoyl‐2‐phenylbenzofuran (2a). Figure S6. 600 MHz 1H NMR spectrum and 151 MHz 13C NMR spectrum of 6‐benzoyl‐2‐phenylbenzofuran (3a). Figure S7. 600 MHz 1H NMR in CDCl3 spectrum and NOESY‐1D spectrum obtained by irradiating the signal of the hydrogen in position 3d of 6‐benzoyl‐2‐phenylbenzofuran (3a). Figure S8. 600 MHz 1H NMR spectrum and 151 MHz 13C NMR spectrum of 3‐benzoyl‐2‐phenyl(d5)benzofuran (1ad 5). Figure S9. 600 MHz 1H NMR spectrum and 151 MHz 13C NMR spectrum of 4‐benzoyl‐2‐phenyl(d5)benzofuran (2ad 5). Figure S10. 600 MHz 1H NMR spectrum and 151 MHz 13C NMR spectrum of 6‐benzoyl‐2‐phenyl(d5)benzofuran (3ad 5). Figure S11. 600 MHz 1H NMR spectrum and 151 MHz 13C NMR spectrum of 4‐(4‐nitrobenzoyl)‐2‐phenylbenzofuran (2b). Figure S12. Top: 600 MHz COSY spectra of 4‐(4‐nitrobenzoyl)‐2‐phenylbenzofuran (2b). Bottom: 600 MHz 1H NMR in CDCl3 and NOESY‐1D spectra obtained by irradiating the signal of the hydrogen in position 3d of 2b. Figure S13. 600 MHz 1H NMR spectrum and 151 MHz 13C NMR spectrum of 6‐(4‐nitrobenzoyl)‐2‐phenylbenzofuran (3b). Figure S14. 600 MHz 1H NMR in CDCl3 and NOESY‐1D spectra obtained by irradiating the signal of the hydrogen in position 3d of 6‐(4‐nitrobenzoyl)‐2‐phenylbenzofuran (3b). Figure S15. 600 MHz 1H NMR spectrum and 151 MHz 13C NMR spectrum of 4‐benzoyl‐2‐(4‐nitrophenyl)benzofuran (2c). Figure S16. Top: 600 MHz COSY spectra of 4‐benzoyl‐2‐(4‐nitrophenyl)benzofuran (2c). Bottom: 600 MHz 1H NMR in CDCl3 and NOESY‐1D spectra obtained by irradiating the signal of the hydrogen in position 3d of 2c. Figure S17. 60 [file RCM-35-e9082-s001.docx]

**Supplementary Material**

**GC-MS^n^ analysis of the crude reaction mixtures from Friedel Craft acylation: unambiguous identification and differentiation of 3-aroylbenzofurans from their 4- and 6-regioisomers**

*Michela Begala,^a,^*^,#^ Michele Mancinelli,^b^ Elias Quezada^c^ and Giovanna Lucia Delogu^a^*

*^a^Department of Life and Environmental Sciences, University of Cagliari, Cittadella Universitaria, s.p.8, 09042 Monserrato (Cagliari), Italy*

*^b^Department of Industrial Chemistry "Toso Montanari", University of Bologna, Viale del Risorgimento, 4, 40136 Bologna, Italy*

*^c^Department of Organic Chemistry, University of Santiago de Compostela,15782 Santiago de Compostela, Spain*

^*^Correspondence to Michela Begala, Department of Life and Environmental Sciences, University of Cagliari, Cittadella Universitaria, s.p.8, 09042 Monserrato (Cagliari), Italy. Email: michelabegala@unica.it

^#^Supervisor and principal investigator

**Materials and methods**

Starting materials, solvent and reagents were obtained from commercial suppliers (Sigma-Aldrich) and were used without further purification. Analytical thin layer chromatography (TLC) was carried out on silica gel 60 F254 plates (0.25 mm), visualized by exposure to UV light. Column chromatography purifications were performed using Aldrich silica gel (60-120) mesh size. Melting points were determined on a Stuart Scientific SMP 11 melting point apparatus and are uncorrected. Concentration and evaporation of the solvent after reaction or extraction were carried out on a rotary evaporator (Büchi Rotavapor) operating at reduced pressure.

^1^H NMR and ^13^C NMR spectra were recorded using a spectrometer (Varian INOVA) operating at a field of 14.4 T (600 MHz for ^1^H, 150.8 MHz for ^13^C) using CDCl_3_ as solvent. Chemical shifts are reported in ppm (δ) relative to TMS (tetramethylsilane) as an internal standard. The 150.8 MHz ^13^C spectra were acquired under proton decoupling conditions with a 36000 Hz spectral width, 5.5 µs (60° tip angle) pulse width, 1 s acquisition time and 4 s delay time. The long relaxation time was needed to observe some quaternary carbons. Coupling constants *J* are expressed in hertz (Hz). Spin multiplicities are given as s (singlet), d (doublet), dd (doublet of doublets), m (multiplet) and apparent triplet (app t). Full assignments of the ^1^H and ^13^C signals were obtained by bi-dimensional experiments (g-COSY, edited-gHSQC,^[1]^ and gHMBC^[2]^ sequences). The NOE experiments were obtained by means of the DPFGSE-NOE^[3]^ sequence. To selectively irradiate the desired signal, a 50 Hz wide shaped pulse was calculated with a refocusing SNOB shape^[4]^ and a pulse width of 37 ms. Mixing time was set to 1.5 s.

***General procedure for the Friedel-Crafts acylation of 2-phenylbenzofurans.***

To a solution of the proper 2-phenylbenzofuran (1mmol) and acyl chloride (1.2 mmol) in anhydrous CH_2_Cl_2_ (2 mL), AlCl_3_ (2 mmol) was added under argon. The reaction mixture was then stirred at 40 °C under argon until the reaction was complete (1-3 h). The reaction was quenched by addition of crushed ice and the mixture was stirred for one more hour. The resulting solution was diluted with water and extracted three times with CH_2_Cl_2_. The combined organic layers were dried over anhydrous MgSO_4_ and concentrated under vacuum. The mixture containing the three reaction products was purified by silica gel chromatography (petroleum ether/ethyl acetate 9:1) to give the pure 3- and 6-benzoyl regioisomers **1a**, **3a**, **1b**, **3b**, **1c**, **3c**. 4-Benzoyl regioisomers **2a**, **2a*d_5_*** **2b** and **2c** were further purified by HPLC. HPLC column Luna 5µ C18(2) 100 Å, flow 5 mL/min, eluent ACN:H_2_O 90:10. NMR spectra and GC-MS analysis of 3-benzoyl isomers **1a**, **1b** and **1c** matched that previously reported by us.^[5-6]^

*3-Benzoyl-2-phenylbenzofuran* (**1a**)*, 4-benzoyl-2-phenylbenzofuran* (**2a**) *and 6-benzoyl-2-phenylbenzofuran* (**3a**)*.*

The mixture of isomers **1a-3a** was obtained by the reaction of 2-phenylbenzofuran with benzoyl chloride. Compound **1a**:^[1]^ white solid; yield: 44 %; mp: 78-80 °C; ^1^H NMR (500 MHz, CDCl_3_, TMS): δ = 7.88 - 7.83 (m, 2H), 7.59 (d, *J* = 8.3 Hz, 1H), 7.60 - 7.55 (m, 2H), 7.52 - 7.44 (m, 2H), 7.39 - 7.27 (m, 6H) ppm; ^13^C NMR (125 MHz, CDCl_3_, TMS): δ = 192.32, 157.81, 154.03, 149.12, 137.92, 133.33, 129.94, 128.61, 128.51, 125.54, 123.94, 121.73, 116.32, 111.44 ppm; MS (EI, 70 eV) *m/z* (%): 298 (100) [M^+.^], 297 (70), 221 (54), 105 (35). Compound **2a**: white solid; yield: 5 %; mp: 71-73 °C; ^1^H NMR (600 MHz, CDCl_3_, 7.26 ppm): δ = 7.93 - 7.88 (m, 2H), 7.88 - 7.83 (m, 2H), 7.73 (dd, *J* = 8.1, 1.0 Hz, 1H), 7.65 - 7.59 (m, 1H), 7.57 (dd, *J* = 7.6, 0.9 Hz, 1H), 7.54 - 7.44 (m, 5H), 7.42 - 7.36 (m, 1H), 7.34 (dd, *J* = 8.1, 7.6 Hz, 1H) ppm; ^13^C NMR (151 MHz, CDCl_3_, 77.0 ppm): δ = 196.37, 158.06, 155.40, 138.48, 132.31, 129.97, 129.95, 129.85, 129.82, 129.12, 128.86, 128.31, 126.83, 125.29, 123.11, 115.14, 102.14 ppm;. MS (EI, 70eV) *m/z* (%): 298 (100) [M^+.^], 221 (74), 105 (25). Compound **3a**: white solid; yield: 36 %; mp: 130-132 °C; ^1^H NMR (600 MHz, CDCl_3_, 7.26 ppm): δ = 8.01 (d, *J* = 1.5 Hz, 1H), 7.92 - 7.87 (m, 2H), 7.86 - 7.81 (m, 2H), 7.78 (dd, *J* = 8.1, 1.4 Hz, 1H), 7.66 (d, *J* = 8.1 Hz, 1H), 7.63 - 7.58 (m, 1H), 7.54 - 7.45 (m, 4H), 7.44 - 7.38 (m, 1H), 7.10 (d, *J* = 1.0 Hz, 1H) ppm; ^13^C NMR (151 MHz, CDCl_3_, 77.0 ppm): δ = 196.26, 159.19, 154.16, 138.23, 133.59, 133.38, 132.08, 129.93, 129.78, 129.37, 128.92, 128.24, 125.39, 125.29, 120.45, 113.57, 101.34 ppm; MS (EI, 70 eV) *m/z* (%): 298 (100) [M^+.^], 221 (89), 105 (19).

Deuterium labeled isomers, **1a*d_5_*-3a*d_5_***, were obtained by 2-phenylbenzofuran and benzoyl chloride-*d*_5_ using the same procedure of **1a-3a**. Compound **1a*d_5_***: white solid; yield: 16 %; mp: 85-87 °C; ^1^H NMR (600 MHz, CDCl_3_, 7.26 ppm): δ = 7.86 - 7.82 (m, 2H), 7.59 (d, *J* = 8.3 Hz, 1H), 7.55 (dd, *J* = 7.8, 1.2 Hz, 1H ), 7.52 -7.46 (m, 1H), 7.40 - 7.30 (m, 3H), 7.29 - 7.24 (m, 1H) ppm; ^13^C NMR (151 MHz, CDCl_3_, 77.0 ppm): δ = 192.37, 157.66, 153.81, 137.79, 133.15, 129.83, 129.24, 129.05, 128.45, 128.39, 127.87, 155.33, 123.82, 121.48, 116.13, 111.22 ppm; MS (EI, 70 eV) *m/z* (%): 303 (100) [M^+.^], 301 (35), 226 (54), 105 (35). Compound **2a*d_5_***: white solid; yield: 7 %; mp: 74-76 °C; ^1^H NMR (600 MHz, CDCl_3_, 7.26 ppm): δ **=** 7.87 - 7.84 (m, 2H), 7.73 (dd, *J* = 8.2, 1.0 Hz, 1H), 7.65 - 7.59 (m, 1H), 7.57 (dd, *J* = 7.6, 0.9 Hz, 1H), 7.54 - 7.45 (m, 3H), 7.34 (appt, *J* = 7.9 Hz, 1H) ppm; ^13^C NMR (151 MHz, CDCl_3_, 77.0 ppm): δ = 196.37, 158.06, 155.40, 138.48, 132.31, 129.96, 129.85, 129.83, 129.81, 128.35, 128.32, 126.83, 124.89, 123.10, 115.13, 102.13 ppm; MS (EI, 70 eV) *m/z* (%): 303 (100) [M^+.^], 226 (75), 105 (20). Compound **3a*d_5_***: white solid; yield: 45%; mp: 138-140 °C; ^1^H NMR (600 MHz, CDCl_3_, 7.26 ppm): δ = 8.00 (d, *J* = 1.4 Hz, 1H), 7.86 - 7.81 (m, 2H), 7.78 (dd, *J* = 8.1, 1.4 Hz, 1H), 7.66 (d, *J* = 8.1 Hz, 1H), 7.64 - 7.58 (m, 1H), 7.51 (dd, *J* = 8.4, 7.0 Hz, 2H), 7.10 (d, *J* = 0.9 Hz, 1H) ppm; ^13^C NMR (151 MHz, CDCl_3_, 77.0 ppm): δ = 196.28, 159.19, 154.16, 138.24, 133.59, 133.40, 132.09, 129.94, 129.62, 128.86, 128.42, 128.25, 125.39, 124.89, 120.45, 113.58, 101.33 ppm; MS (EI, 70 eV) *m/z* (%): 303 (100) [M^+.^], 226 (88), 105 (20).

*3-(4-Nitrobenzoyl)-2-phenylbenzofuran* (**1b**), *4-(4-nitrobenzoyl)-2-phenylbenzofuran* (**2b**) and *6-(4-nitrobenzoyl)-2-phenylbenzofuran* (**3b**).

The mixture of isomers **1b-3b** was obtained by the reaction of 2-phenylbenzofuran with 4-nitrobenzoyl chloride. Compound **1b**^[2]^: yellow solid; yield: 58 %; mp: 126-128 °C; ^1^H NMR (600 MHz, CDCl_3_, 7.26 ppm): δ = 7.89 (d, *J* = 8.22 Hz, 2H), 7.71 (d, *J* = 8.02 Hz, 1H), 7.61 (d, *J* = 8.29 Hz, 1H), 7.57 (d, *J* = 8.16 Hz, 2H), 7.42 (t, *J* = 7.72 Hz, 1H), 7.35 - 7.31 (m, 2H), 7.27 (t, *J* = 7.43 Hz, 2H), 7.10 (d, *J* = 8.22 Hz, 2H) ppm; ^13^C NMR (151 MHz, CDCl_3_, 77.0 ppm): δ = 190.23, 159.71, 154.05, 149.93, 142.92, 130.54, 130.45, 129.04, 128.91, 128.52, 127.73, 125.94, 124.31, 123.40, 121.53, 115.52, 111.44 ppm; MS (EI, 70 eV) *m/z* (%): 343 (100) [M^+.^], 221 (43), 150 (5). Compound **2b**: yellow solid; yield: 6 %; mp: 118-120 °C; ^1^H NMR (600 MHz, CDCl_3_, 7.26 ppm): δ = 8.40 - 8.35 (m, 2H), 8.00 - 7.95 (m, 2H), 7.95 - 7.91 (m, 2H), 7.79 (dt, *J* = 8.2, 0.9 Hz, 1H), 7.59 (d, *J* = 1.0 Hz, 1H), 7.52 - 7.46 (m, 3H), 7.45 - 7.39 (m, 1H), 7.35 (t, *J* = 7.9 Hz, 1H) ppm; ^13^C NMR (151 MHz, CDCl_3_, 77.0 ppm): δ 194.48, 158.95, 155.51, 149.73, 143.96, 130.56, 130.02, 129.69, 129.46, 128.95, 128.41, 127.27, 125.42, 123.57, 123.22, 116.20, 102.04 ppm; MS (EI, 70 eV) *m/z* (%): 343 (100) [M^+.^], 221 (50), 150 (2). Compound **3b**: yellow solid; yield: 59 %; mp: 170-172 °C; ^1^H NMR (600 MHz, CDCl_3_, 7.26 ppm): δ = 8.39 - 8.34 (m, 2H), 8.00 - 7.93 (m, 3H), 7.93 - 7.88 (m, 2H), 7.75 (dd, *J* = 8.1, 1.4 Hz, 1H), 7.69 (d, *J* = 8.1 Hz, 1H), 7.52 - 7.46 (m, 2H), 7.46 - 7.40 (m, 1H), 7.12 (s, 1H) ppm; ^13^C NMR (151 MHz, CDCl_3_, 77.0 ppm): δ 194.26, 159.96, 154.18, 149.63, 143.73, 134.37, 132.18, 130.53, 129.68, 129.51, 129.00, 125.40, 125.36, 123.53, 120.82, 113.53, 101.37 ppm; MS (EI, 70 eV) *m/z* (%): 343 (100) [M^+.^], 221 (55), 150 (1).

*3-Benzoyl-2-(4-nitrophenyl)benzofuran* (**1c**), *4-benzoyl-2-(4-nitrophenyl)benzofuran* (**2c**) and *6-benzoyl-2-(4-nitrophenyl)benzofuran* (**3c**).

The mixture of isomers **1c-3c** was obtained by the reaction of 2-(4-nitrophenyl)benzofuran with benzoyl chloride. Compound **1c**:^[2]^ yellow solid; yield: 7%; mp: 148-150 °C; ^1^H NMR (600 MHz, CDCl_3_, 7.26 ppm): δ = 8.18 (d, *J* = 8.73 Hz, 2H), 7.93 (d, *J* = 8.73 Hz, 2H), 7.89 (d, *J* = 7.52 Hz, 2H), 7.63 (d, *J* = 8.26 Hz, 1H), 7.58 (t, *J* = 7.47 Hz, 1H), 7.47 - 7.39 (m, 4H), 7.28 (t, *J* = 7.66 Hz, 1H) ppm; ^13^C NMR (151 MHz, CDCl_3_, 77.0 ppm): δ = 191.82, 154.15, 153.73, 147.82, 137.34, 135.31, 134.05, 129.93, 128.82, 128.61, 128.15, 126.53, 124.34, 123.71, 121.83, 119.04, 111.53 ppm; MS (EI, 70 eV) *m/z* (%): 343 (100) [M^+.^], 266 (50), 105 (25). Compound **2c**: yellow solid; yield: 39 %; mp: 155-157 °C; ^1^H NMR (600 MHz, CDCl_3_, 7.26 ppm): δ = 8.36 - 8.31 (m, 2H), 8.08 - 8.03 (m, 2H), 7.88 - 7.82 (m, 2H), 7.78 (dt, *J* = 8.2, 1.0 Hz, 1H), 7.74 (d, *J* = 1.0 Hz, 1H), 7.67 - 7.60 (m, 2H), 7.56 - 7.50 (m, 2H), 7.44 (d, *J* = 7.6 Hz, 1H) ppm; ^13^C NMR (151 MHz, CDCl_3_, 77.0 ppm): δ = 196.04 , 155.91, 155.23, 147.63, 138.12, 135.78, 132.57, 130.54, 129.97, 129.11, 128.42, 127.32, 125.67, 124.64, 124.37, 115.49, 105.79 ppm; MS (EI, 70 eV) *m/z* (%): 343 (100) [M^+.^], 266 (75), 105 (25). Compound **3c**: yellow solid; yield: 13 %; mp: 210-212 °C; ^1^H NMR (600 MHz, CDCl_3_, 7.26 ppm): δ = 8.37 - 8.32 (m, 2H), 8.08 - 8.04 (m, 2H), 8.03 (d, J = 1.3 Hz, 1H), 7.86 - 7.82 (m, 2H), 7.81 (dd, *J* = 8.1, 1.3 Hz, 1H), 7.73 (d, *J* = 8.1, 1H), 7.62 (ddt, *J* = 8.7, 6.9, 1.3 Hz, 1H), 7.52 (dd, *J* = 8.5, 7.1 Hz, 2H), 7.32 (s, 1H) ppm; ^13^C NMR (151 MHz, CDCl_3_, 77.0 ppm): δ = 196.02, 156.24, 154.72, 147.78, 137.87, 135.54, 135.03, 132.51, 132.38, 129.98, 128.35, 125.72, 125.70, 124.40, 121.27, 113.80, 104.89 ppm; MS (EI, 70 eV) *m/z* (%): 343 (100) [M^+.^], 266 (98), 105 (25).

*[2 x ^18^O]-Benzoic acid*.^[7]^

White solid: mp: 123-125 ºC; MS (EI, 70eV) *m/z* (%): 126 (100) [2 x ^18^O] M^+.^, 124 (5) [^18/16^O] M^+.^, 122 (1) [2 x ^16^O] M^+.^, 107 (70), 77 (45).

*[^18^O]-Benzoyl chloride*.^[7]^

[2x ^18^O]-Benzoic acid (0.317 g, 2.5 mmol) was dissolved in dichloromethane dry (5 mL). Thionyl chloride (0.6 mL, 8 mmol) and 2 mL of DCM were added at 0 °C. The reaction mixture was then stirred at room temperature and monitored by TLC (petroleum ether/ethyl acetate 10:1) and GC/MS. After the reaction was completed (2 h), the solvent was removed under reduced pressure to give the [^18^O]-benzoyl chloride as a light yellow oil with 90% yield. MS (EI, 70 eV) *m/z* (%): 142 (2) [^18^O] M^+.^, 107 (100), 77 (20).

*3-[^18^O]-(Benzoyl)-2-(4-nitrophenyl)benzofuran* (**1c[^18^O]**).

Treatment of 2-phenylbenzofuran with [^18^O]-benzoyl chloride, as described for **1c**, gave **1c[^18^O]** with identical physical and spectral properties except MS. MS (EI, 70 eV) *m/z* (%): 345 (100) [^18^O] M^+.^, 268 (29), 107 (10).

References

[1] (a) Bradley, S. A.; Krishnamurthy, K. *Magn. Reson. Chem.* **2005**, *43*, 117; (b) Willker, W.; Leibfritz, D.; Kerssebaum, R.; Bermel, W. *Magn. Reson. Chem.* **1993**, *31*, 287.

[2] Hurd, R. E.; John, B. K. J. *Magn. Reson.* **1991**, *91*, 648.

[3] (a) Stott, K.; Stonehouse, J.; Keeler, J.; Hwand, T.-L.; Shaka, A. J. *J. Am. Chem. Soc.* **1995**, *117*, 4199; (b) Stott, K.; Keeler, J.; Van, Q. N.; Shaka, A. J. J. *Magn. Reson.* **1997**, *125*, 302; (c) Van, Q. N.; Smith, E. M.; Shaka, A. J. J. *Magn. Reson.* **1999**, *141*, 191; (d) See also: Claridge, T. D. W. High Resolution NMR Techniques in Organic Chemistry; Pergamon: Amsterdam, 1999.

[4] Kupce, E.; Boyd, J.; Campbell, I. D. J. *Magn. Reson.*, Ser. B **1995**, *106*, 300.

[5] Begala, M.; Caboni, P. L.; Matos, M. J.; Delogu, G. L. *Tetrahedron Lett.* **2018**, *59* (18), 1711-1714.

[6] Begala, M.; Mancinelli, M.; Delogu, G. L*. Tetrahedron Lett.* **2020**, *61*, 151634.

[7] Stanislaw F. Wnuk et al. *J. Org. Chem*., **2002**, 67, 1816-1819.

**Figure S1.** EI mass spectra of labelled regioisomers **1a*d_5_*** (A), **2a*d_5_*** (B), and **3a*d_5_*** (C).

**Figure S2.** MS/MS mass spectra of the ions at *m/z* 303 from labeled **1a*d_5_*** (A), **2a*d_5_*** (B), and **3a*d_5_*** (C).

**Figure S3.** EI mass spectra of [2 x ^18^O]-benzoic acid (A), and of [^18^O]-benzoyl chloride (B).

**Scheme S1.** Proposed mechanisms of elimination of radical OH^.^ from labeled **1c[^18^O]**.

**2a**

**Figure S4.** 600 MHz ^1^H NMR spectrum and 151 MHz ^13^C NMR spectrum of 4-benzoyl-2-phenylbenzofuran (**2a**).

**Figure S5.** HSQC and COSY spectra of 4-benzoyl-2-phenylbenzofuran (**2a**).

**3a**

**Figure S6.** 600 MHz ^1^H NMR spectrum and 151 MHz ^13^C NMR spectrum of 6-benzoyl-2-phenylbenzofuran (**3a**).

**3a**

**Figure S7.** 600 MHz ^1^H NMR in CDCl_3_ spectrum and NOESY-1D spectrum obtained by irradiating the signal of the hydrogen in position 3d of 6-benzoyl-2-phenylbenzofuran (**3a**).

**1a*d_5_***

**Figure S8.** 600 MHz ^1^H NMR spectrum and 151 MHz ^13^C NMR spectrum of 3-benzoyl-2-phenyl(d_5_)benzofuran (**1a*d_5_***).

**2a*d_5_***

**Figure S9.** 600 MHz ^1^H NMR spectrum and 151 MHz ^13^C NMR spectrum of 4-benzoyl-2-phenyl(d_5_)benzofuran (**2a*d_5_***).

**3a*d_5_***

**Figure S10.** 600 MHz ^1^H NMR spectrum and 151 MHz ^13^C NMR spectrum of 6-benzoyl-2-phenyl(d_5_)benzofuran (**3a*d_5_***).

**2b**

**Figure S11.** 600 MHz ^1^H NMR spectrum and 151 MHz ^13^C NMR spectrum of 4-(4-nitrobenzoyl)-2-phenylbenzofuran (**2b**).

**Figure S12.** Top: 600 MHz COSY spectra of 4-(4-nitrobenzoyl)-2-phenylbenzofuran (**2b**). Bottom: 600 MHz ^1^H NMR in CDCl_3_ and NOESY-1D spectra obtained by irradiating the signal of the hydrogen in position 3d of **2b**.


 **3b**

**Figure S13.** 600 MHz ^1^H NMR spectrum and 151 MHz ^13^C NMR spectrum of 6-(4-nitrobenzoyl)-2-phenylbenzofuran (**3b**).

**3b**

**Figure S14.** 600 MHz ^1^H NMR in CDCl_3_ and NOESY-1D spectra obtained by irradiating the signal of the hydrogen in position 3d of *6-(4-nitrobenzoyl)-2-phenylbenzofuran* (**3b**).

**2c**

**Figure S15.** 600 MHz ^1^H NMR spectrum and 151 MHz ^13^C NMR spectrum of 4-benzoyl-2-(4-nitrophenyl)benzofuran (**2c**).

**Figure S16.** Top: 600 MHz COSY spectra of 4-benzoyl-2-(4-nitrophenyl)benzofuran (**2c**). Bottom: 600 MHz ^1^H NMR in CDCl_3_ and NOESY-1D spectra obtained by irradiating the signal of the hydrogen in position 3d of **2c**.

**3c**

**Figure S17.** 600 MHz ^1^H NMR spectrum and 151 MHz ^13^C NMR spectrum of 6-benzoyl-2-(4-nitrophenyl)benzofuran (**3c**).

**Figure S18.** 600 MHz ^1^H NMR in CDCl_3_ and NOESY-1D spectra obtained by irradiating the signal of the hydrogen in position 3d of 6-benzoyl-2-(4-nitrophenyl)benzofuran (**3c**).

**Table S1.** Repeatability of relative peak intensity of ions in MS^2^ spectrum of **1a**

| *m/z* | Average relative intensity (%) | SD (%) | RSD (%) |
| --- | --- | --- | --- |
| 297 | 100 | 0 | 0 |
| 269 | 3.8 | 0.45 | 11.77 |

**Table S2.** Repeatability of relative peak intensity of ions in MS^3^ spectra of **1a**

| *m/z* | Average relative intensity (%) | SD (%) | RSD (%) |
| --- | --- | --- | --- |
| 193 | 35.6 | 2.41 | 6.76 |
| 165 | 100 | 0 | 0 |

**Table S3.** Repeatability of relative peak intensity of ions in MS^2^ spectrum of **2a**

| *m/z* | Average relative intensity (%) | SD (%) | RSD (%) |
| --- | --- | --- | --- |
| 221 | 100 | 0 | 0 |
| 193 | 3.2 | 0.44 | 13.98 |

**Table S4.** Repeatability of relative peak intensity of ions in MS^3^ s spectrum of **2a**

| *m/z* | Average relative intensity (%) | SD (%) | RSD (%) |
| --- | --- | --- | --- |
| 193 | 100 | 0 | 0 |
| 165 | 14.4 | 0.89 | 6.21 |

**Table S5.** Repeatability of relative peak intensity of ions in MS^2^ spectrum of **3a**

| *m/z* | Average relative intensity (%) | SD (%) | RSD (%) |
| --- | --- | --- | --- |
| 221 | 100 | 0 | 0 |

**Table S6.** Repeatability of relative peak intensity of ions in MS^3^ spectrum of **3a**

| *m/z* | Average relative intensity (%) | SD (%) | RSD (%) |
| --- | --- | --- | --- |
| 193 | 100 | 0 | 0 |
| 165 | 28.0 | 2.74 | 9.78 |

**Table S7.** Repeatability of relative peak intensity of ions in MS^2^spectrum of **1b**

| *m/z* | Average relative intensity (%) | SD (%) | RSD (%) |
| --- | --- | --- | --- |
| 342 | 100 | 0 | 0 |
| 312 | 24.6 | 2.19 | 8.91 |
| 296 | 70.0 | 6.12 | 8.75 |

**Table S8.** Repeatability of relative peak intensity of ions in MS^3^ spectra of **1b**

| *m/z* | Average relative intensity (%) | SD (%) | RSD (%) |
| --- | --- | --- | --- |
| 193 | 42.4 | 1.14 | 2,69 |
| 165 | 100 | 0 | 0 |

**Table S9.** Repeatability of relative peak intensity of ions in MS^2^ spectrum of **2b**

| *m/z* | Average relative intensity (%) | SD (%) | RSD (%) |
| --- | --- | --- | --- |
| 296 | 2.8 | 0.44 | 15.97 |
| 221 | 100 | 0 | 0 |
| 193 | 17.4 | 0.89 | 5.14 |

**Table S10.** Repeatability of relative peak intensity of ions in MS^3^ spectrum of **2b**

| *m/z* | Average relative intensity (%) | SD (%) | RSD (%) |
| --- | --- | --- | --- |
| 193 | 100 | 0 | 0 |
| 165 | 14.8 | 1.09 | 7.40 |

**Table S11.** Repeatability of relative peak intensity of ions in MS^2^ spectrum of **3b**

| *m/z* | Average relative intensity (%) | SD (%) | RSD (%) |
| --- | --- | --- | --- |
| 296 | 7.4 | 0.89 | 12.09 |
| 221 | 100 | 0 | 0 |
| 193 | 10.8 | 1.3 | 12.07 |

**Table S12.** Repeatability of relative peak intensity of ions in MS^3^ spectrum of **3b**

| *m/z* | Average relative intensity (%) | SD (%) | RSD (%) |
| --- | --- | --- | --- |
| 193 | 100 | 0 | 0 |
| 165 | 21.2 | 1.79 | 8.44 |

**Table S13.** Repeatability of relative peak intensity of ions in MS^2^ spectrum of **1c**

| *m/z* | Average relative intensity (%) | SD (%) | RSD (%) |
| --- | --- | --- | --- |
| 342 | 100 | 0 | 0 |
| 326 | 29.2 | 1.09 | 3.75 |
| 296 | 9.0 | 0.71 | 7.86 |
| 266 | 7.4 | 0.89 | 12.09 |
| 210 | 7.0 | 0.71 | 10.10 |

**Table S14.** Repeatability of relative peak intensity of ions in MS^3^ spectrum of **1c**

| *m/z* | Average relative intensity (%) | SD (%) | RSD (%) |
| --- | --- | --- | --- |
| 236 | 14.8 | 0.44 | 3.24 |
| 220 | 100 | 0 | 0 |
| 208 | 20.4 | 0.54 | 2.68 |

**Table S15.** Repeatability of relative peak intensity of ions in MS^2^ spectrum of **2c**

| *m/z* | Average relative intensity (%) | SD (%) | RSD (%) |
| --- | --- | --- | --- |
| 296 | 3.8 | 0.44 | 11.77 |
| 266 | 100 | 0 | 0 |

**Table S16.** Repeatability of relative peak intensity of ions in MS^3^ spectrum of **2c**

| *m/z* | Average relative intensity (%) | SD (%) | RSD (%) |
| --- | --- | --- | --- |
| 238 | 82.6 | 0.89 | 1.08 |
| 220 | 100 | 0 | 0 |
| 210 | 14.6 | 0.89 | 6.13 |
| 208 | 28.0 | 1.22 | 4.37 |

**Table S17.** Repeatability of relative peak intensity of ions in MS^2^ spectrum of **3c**

| *m/z* | Average relative intensity (%) | SD (%) | RSD (%) |
| --- | --- | --- | --- |
| 326 | 4.8 | 0.44 | 9.32 |
| 296 | 17.6 | 1.51 | 8.62 |
| 266 | 100 | 0 | 0 |

**Table S18.** Repeatability of relative peak intensity of ions in MS^3^ spectrum of **3c**

| *m/z* | Average relative intensity (%) | SD (%) | RSD (%) |
| --- | --- | --- | --- |
| 238 | 15.8 | 0.44 | 2.83 |
| 220 | 100 | 0 | 0 |
| 210 | 3 | 0 | 0 |
| 208 | 20.4 | 0.54 | 2.68 |
